# Feasibility of Localized Metabolomics in the Study of Pancreatic Islets and Diabetes

**DOI:** 10.3390/metabo9100207

**Published:** 2019-09-29

**Authors:** Oscar Alcazar, Luis F. Hernandez, Ashley Tschiggfrie, Michael J. Muehlbauer, James R. Bain, Peter Buchwald, Midhat H. Abdulreda

**Affiliations:** 1 Diabetes Research Institute and Cell Transplant Center, University of Miami Miller School of Medicine, Miami, FL 33136, USA; o.alcazar@med.miami.edu (O.A.); lfh34@med.miami.edu (L.F.H.); axt939@med.miami.edu (A.T.); 2 Duke Molecular Physiology Institute, Duke University Medical Center, Durham, NC 27708, USA; michael.muehlbauer@duke.edu (M.J.M.); james.bain@duke.edu (J.R.B.); 3 Department of Molecular and Cellular Pharmacology, University of Miami Miller School of Medicine, Miami, FL 33136, USA; 4 Department of Surgery, University of Miami Miller School of Medicine, Miami, FL 33136, USA; 5 Department of Microbiology and Immunology, University of Miami Miller School of Medicine, Miami, FL 33136, USA; 6 Department of Ophthalmology, University of Miami Miller School of Medicine, Miami, FL 33136, USA

**Keywords:** autoimmune diabetes, anterior chamber of the eye, gender differences, non-obese diabetic (NOD) mice, pancreatic islet, local metabolomics, non-targeted metabolomics, GC-MS, islet microenvironment, T1D biomarkers

## Abstract

(1) Background: Disruption of insulin production by native or transplanted pancreatic islets caused by auto/allo-immunity leads to hyperglycemia, a serious health condition and important therapeutic challenge due to the lifelong need for exogeneous insulin administration. Early metabolic biomarkers can prompt timely interventions to preserve islet function, but reliable biomarkers are currently lacking. We explored the feasibility of “localized metabolomics” where initial biomarker discovery is made in aqueous humor samples for further validation in the circulation. (2) Methods: We conducted non-targeted metabolomic studies in parallel aqueous humor and plasma samples from diabetic and nondiabetic mice. Metabolite levels and associated pathways were compared in both compartments as well as to an earlier longitudinal dataset in hyperglycemia-progressor versus non-progressor non-obese diabetic (NOD) mice. (3) Results: We confirmed that aqueous humor samples can be used to assess metabolite levels. About half of the identified metabolites had well-correlated levels in the aqueous humor and plasma. Several plasma metabolites were significantly different between diabetic and nondiabetic animals and between males and females, and many of them were correlated with the aqueous humor. (4) Conclusions: This study provides proof-of-concept evidence that aqueous humor samples enriched with islet-related metabolites and representative of the immediate islet microenvironment following intraocular islet transplant can be used to assess metabolic changes that could otherwise be overlooked in the general circulation. The findings support localized metabolomics, with and without intraocular islet transplant, to identify biomarkers associated with diabetes and islet allograft rejection.

## 1. Introduction

Blood glucose levels are maintained in relatively narrow ranges that are somewhat species-specific. These narrow ranges are achieved by the fine-tuned function of the insulin-producing cells within the pancreatic islets (or equivalents) in response to changes in systemic glucose levels [1]. In humans, the destruction of the insulin-producing β-cells by an immune attack, as occurs in type 1 diabetes (T1D), is a life-threating problem that leads to serious health complications associated with chronic hyperglycemia and a lifelong need for daily exogenous insulin therapy. This particularly represents a therapeutic challenge as, thus far, all clinical trials with immunomodulatory approaches aimed to stop T1D progression and the decline of insulin production have failed [2,3,4,5]. Currently, T1D is the only autoimmune disease without an approved immunomodulatory treatment [5,6]. The incidence rate of T1D worldwide varies significantly; for example, it is estimated at 0.1 per 100,000/year in China or Venezuela versus >40 per 100,000/year in Finland and Sardinia [7,8,9,10]. In the United States, it approximately affects 1 person in every 400–600 children and adolescents (average prevalence of 1.93 per 1000 in 2009) [9]. While the etiology and pathogenic mechanisms that lead to T1D progression and onset of hyperglycemia are complex, an interplay is suspected between various risk factors such as genetic predisposition, environmental factors, and other unknown events [11]. It is now also recognized that metabolic perturbations are involved before manifestation of the clinical disease [12,13,14,15]; hence, highlighting the importance of metabolic biomarkers in early detection and prevention.

Metabolomics has been used to study pancreatic islet physiology and pathophysiology and to investigate the metabolic perturbations associated with T1D in children as well as in animal models – relevant summaries can be found in [12,13,14,15]. The non-obese diabetic (NOD) mice spontaneously develop diabetes in a manner that reproduces many aspects of the human disease and, thus, have been widely used as an animal model of T1D including in metabolomic studies [16,17,18,19,20,21,22]. However, prior studies have often been limited by the need for relatively large amounts of samples for reliable metabolomics, e.g., millions of cells (meaning thousands of islets) per conditions for LC-MS type analyses [14]. Here, we present exploratory non-targeted metabolomic studies in mice to (a) demonstrate proof-of-concept for “localized metabolomics” using small aqueous humor samples that are expected to be enriched with islet-related metabolites and representative of the immediate islet microenvironment following intraocular islet transplant, (b) explore the feasibility of initial biomarker discovery in the enriched local microenvironment for islet/diabetes-specific changes that can also be measured systemically (in plasma), and (c) establish the relevance of locally measured metabolites (in aqueous humor) to systemic changes caused by diabetes. 

We previously demonstrated that aqueous humor samples are representative of the local islet microenvironment following islet transplant in the anterior chamber of the eye (ACE). We also showed that access to these local samples is uniquely enabled by the intraocular islet transplant approach [23]. In addition, we recently showed that intraocular islet transplant allows longitudinal sampling of the islet microenvironment during the progression of T1D in NOD mice [24]. Therefore, longitudinal localized metabolomics enabled by the ACE platform may help the identification of islet-related metabolites enriched in the aqueous humor during development of T1D and, consequently, facilitate the discovery of reliable early predictive biomarkers of T1D that may be overlooked in the general circulation. To our knowledge, localized metabolomics as a concept has been applied so far very rarely and only in a few cases for tissue characterization [25,26,27]. Here, we explore its extension in the ACE platform for the longitudinal study of autoimmune T1D and islet allograft rejection.

## 2. Results

### 2.1. Non-Targeted Metabolomics in Parallel Local (Aqueous Humor) and Systemic (Plasma) Samples

We performed GC-MS-based metabolomics using aqueous humor (both eyes) and parallel plasma samples collected at the same time from each individual mouse (12 male + 12 female C57BL/6 mice and 3 diabetic/progressor + 18 nondiabetic/non-progressor female NOD mice; see Sample Collection in Materials and Methods for further details). To reach adequate sample volumes (~50 µL), the aqueous humor samples were pooled from groups of 4 animals and were compared to the averaged corresponding plasma samples following GC-MS metabolomics data analysis. We identified a total of 122 metabolite features that were present in the majority of samples, which we annotated relative to a reference library, and quantified as log_2_ integrated peak areas. A complete list of the metabolite features is provided in the Supplementary Material (Table S1). Using these data, three different comparisons were made between the aqueous humor versus plasma (in normoglycemic animals), between males versus females (in C57BL/6), and between T1D progressors versus non-progressors (in NOD). To establish the potential relevance of the metabolic pathways corresponding to the identified metabolites in the context of autoimmune T1D and islet transplant models, the results were also compared to a dataset from a previous longitudinal metabolomic study of T1D progressor versus non-progressor NOD mice [28].

#### 2.1.1. Non-Targeted Metabolomics in Aqueous Humor Samples

**Figure 1:**
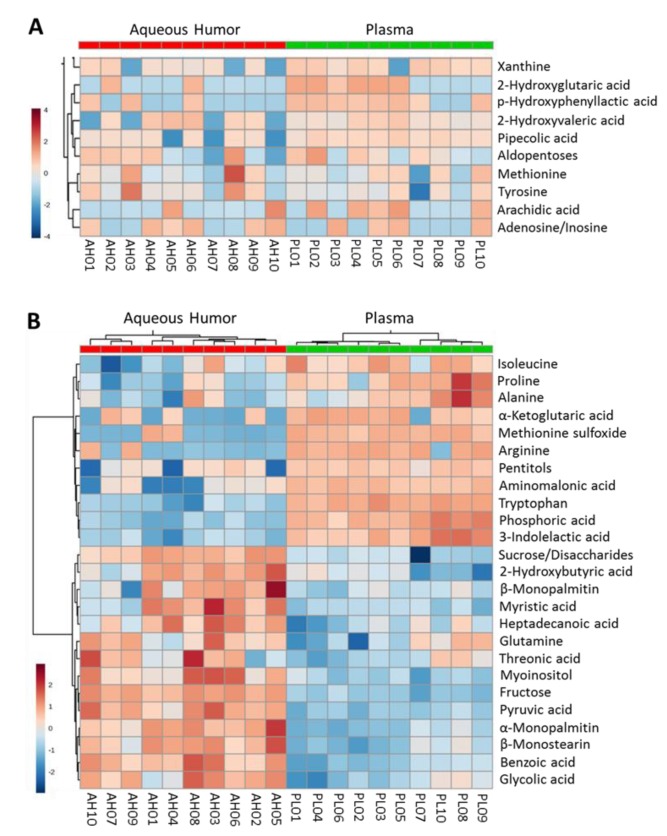
Heatmap analysis of metabolite levels identified in parallel aqueous humor and corresponding plasma samples as measured by gas chromatography mass spectrometry (GC-MS) (log_2_ area under the curve AUC values normalized by median and based on *n* = 10 pooled samples obtained from normoglycemic C57BL/6 and NOD mice). Data shown are for metabolites that are most (**A**) equally and (**B**) differently distributed between the aqueous humor and plasma.

We compared the levels of metabolites identified in the aqueous humor samples (*n* = 10; 6 pools from C57BL/6 and 4 pools from NOD) and the parallel plasma samples for the same animals (averages of the individual plasma measurements corresponding to each aqueous humor pool). As illustrated in the heatmap analysis shown in Figure 1A, several metabolites showed similar levels in both compartments (aqueous humor and plasma). There were also metabolites that showed higher levels (enrichment) in the aqueous humor compared to plasma and vice versa (Figure 1B). A similar comparison, but separated for the two strains (C57BL6, *n* = 6 and NOD, *n* = 4) is included in Supplementary Material, Figure S1 to highlight that while there were differences, there were also many similarities consistent with the pooled data.

**Figure 2:**
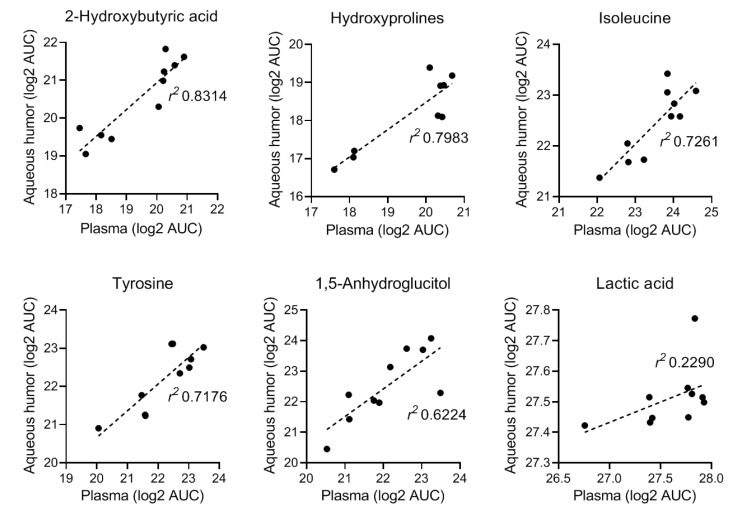
Representative metabolites that showed strong correlation in their aqueous humor and plasma levels as measured by GC-MS. Data shown as log_2_ AUC values (arbitrary units) acquired in aqueous humor and plasma samples (*n* = 10 each).

**Figure 3:**
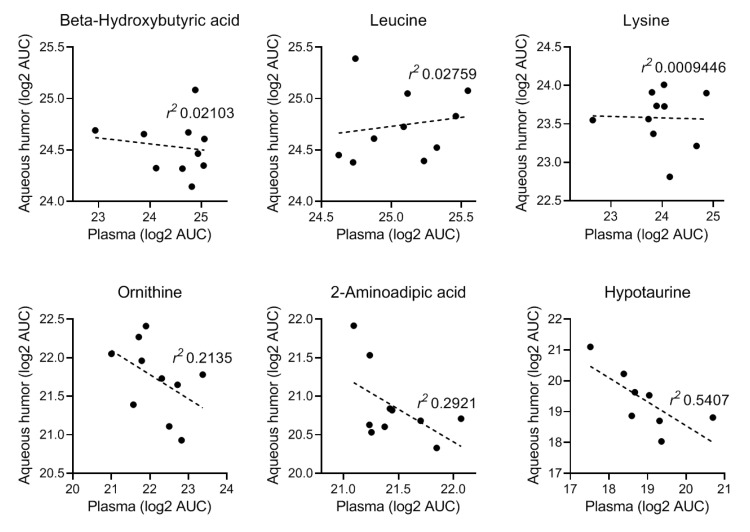
Representative metabolites that showed low or inverse correlation between their aqueous humor and plasma levels as measured by GC-MS. Data shown as log_2_ AUC values (arbitrary units) acquired in aqueous humor and plasma samples (*n* = 10 each).

Of the 88 annotated metabolites with quantifiable correlations in this dataset, we found 41 (47%) that showed good correlation (*r* > 0.3); 37 that showed little correlation (−0.3 ≤ *r* ≤ 0.3), and 10 metabolites that were anti-correlated in the aqueous humor and plasma (*r* < −0.3) (Figure 2 and Figure 3).

**Figure 4:**
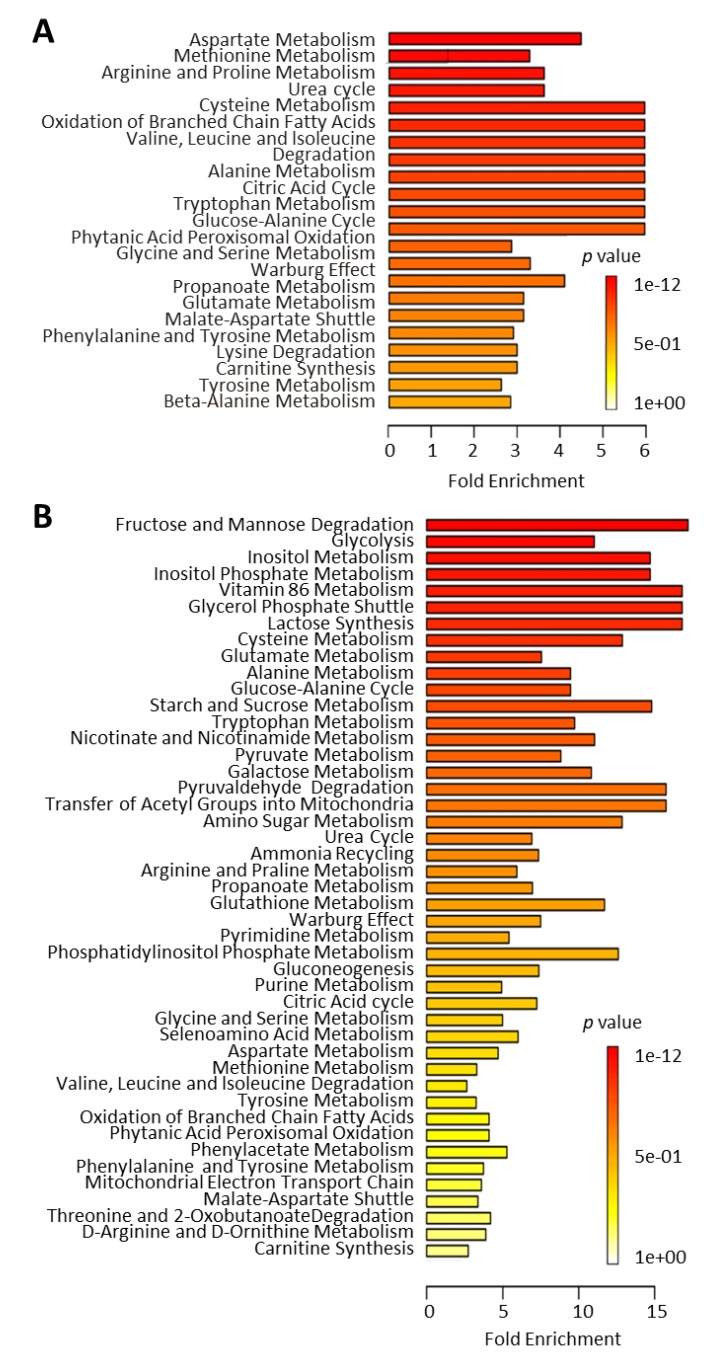
Metabolic pathways associated with metabolite sets that were (**A**) equally distributed or (**B**) significantly enriched in aqueous humor versus plasma samples collected from normoglycemic C57BL/6 and NOD mice (*n* = 10 each). Figures obtained using the quantitative enrichment analysis (QEA) approach of the metabolite set enrichment analysis (MSEA) tool of MetaboAnalyst. Pathways are shown in order of decreasing significance from top to bottom (increasing nominal *p* values, colored from red to yellow) with bars indicating their estimated fold enrichment (see [32] for details).

To highlight the potential relevance of the new information that could be obtained from this novel approach of localized metabolomics, we performed pathway association analysis of these data, i.e., pathways most closely associated with the comparison based on the metabolite levels in both compartments. We performed the analysis (MetaboAnalyst) for metabolites that were equally distributed in the aqueous humor and plasma (Figure 4A) as well as those showing enrichment in aqueous humor (Figure 4B). The analysis showed that the aqueous humor provided information on metabolic pathways that are particularly relevant to pancreatic islet function [29,30,31]. Further, pathway-impact analysis, which combines enrichment and topological (pathway centrality) parameters, was also done on this dataset to compare it with changes caused by T1D onset (see later).

#### 2.1.2. Exploratory Assessment of Diabetes-Induced Changes in the Metabolome

The present dataset allowed preliminary assessment of diabetes-induced changes in the metabolome as measured in the plasma samples collected from hyperglycemia/T1D progressor (*n* = 3) and non-progressor (*n* = 18) NOD mice. The analysis showed significant differences (*p* ≤ 0.05, *t*-test) only for docosahexaenoic acid, 6-deoxyhexose, urea, and ornithine—likely due to the very limited number of diabetic samples. Nevertheless, while these studies were exploratory in nature, they also allowed for a comparison with our prior dataset from a previous longitudinal study in T1D progressor and non-progressor NOD mice [28].

### 2.2. Representative Findings from A Longitudinal NOD Study

Previously, we completed a non-targeted longitudinal metabolomic study in male and female NOD mice (15F + 15M) to characterize the time-profile of the changes in the systemic metabolic landscape caused by T1D progression and the onset of hyperglycemia [28]. This involved collection of a large number of samples (collected at 6, 11, 16, 21, and 26 weeks of age from mice whose glycemia was monitored and retrospectively designated as T1D progressors or non-progressors depending on whether they became hyperglycemic or not by the end of the study). Diabetes onset in these NOD mice was typical and similar to what we have obtained previously [33,34], starting around 12 weeks of age with approximately 60% of females and 30% of males becoming diabetic by week 26 (Figure S2A). At the end of the study (week 26 of age), representative samples from 3F + 3M non-progressors (control) and 4F + 4M T1D progressors were subjected to global metabolomics, which identified and quantified a large number of metabolites (676 and 706 in blood and feces, respectively). A considerable proportion of the metabolites was significantly altered by T1D (hyperglycemia) onset. Figure S2B shows the longitudinal time-profiles of metabolites showing the largest fold-change in T1D versus controls in blood samples at week 26 (representative metabolites with >4-fold change and *p* < 0.05) [28]. Here, we reanalyzed some of those results focusing on metabolic pathways to assess the relevance of the data obtained in the aqueous humor on the local metabolome for islet/diabetes-directed studies.

#### 2.2.1. Metabolic Changes in Plasma of T1D Progressor Versus Non-Progressor NOD Mice

**Figure 5:**
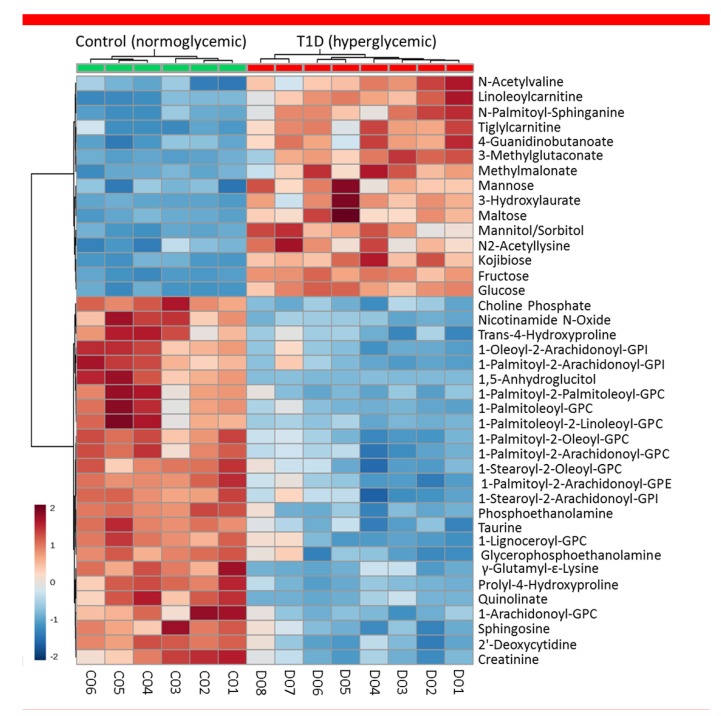
Heatmap analysis highlighting metabolites showing the largest change in plasma between T1D progressor (*n* = 8 mice, 4F + 4M) and non-progressor (control; *n* = 6, 3F + 3M) NOD mice at 26 weeks of age. All progressors were diabetic at the time of this analysis; the duration of diabetes was 5–14 weeks – see Figure S2A for the corresponding rates of onset).

Several metabolites were found at significantly different levels in the plasma of T1D progressors at all time-points as compared to non-progressors, and these differences increased further following onset of hyperglycemia at 26 weeks of age when all progressors were confirmed to be diabetic (Figure S2B). A large proportion of the detected metabolites had significantly different levels in the progressors versus non-progressors (controls); i.e., 57.8% of the 676 metabolites detected in blood (266 increased + 125 decreased = 391 total) and 27.8% of the 706 metabolites detected in feces (82 increased + 114 decreased = 196 total). A heatmap highlighting some of the metabolites showing the largest change in plasma between progressor and non-progressor animals (at 26 weeks of age) is shown in Figure 5. These data clearly illustrated the profound metabolic changes caused by the onset of T1D.

#### 2.2.2. Metabolic Pathways Affected by Hyperglycemia Onset in NOD Mice

**Figure 6:**
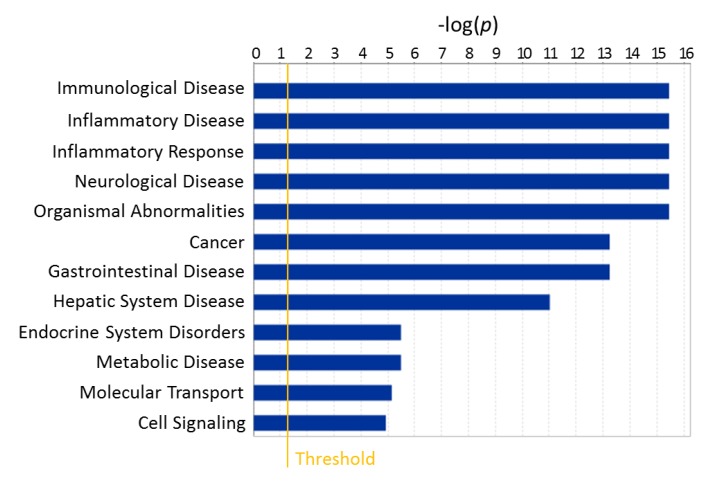
Diseases and functions most affected by onset of T1D in NOD mice using blood data of 524 metabolites measured at 26 weeks of age [28], as identified here by a corresponding enrichment analysis (Ingenuity Pathway Analysis).

The onset of diabetes/hyperglycemia significantly affected several metabolic pathways in progressor NOD mice compared with non-progressor controls from the same batch. Our previous analysis comparing T1D progressors versus non-progressors using data from all time-points identified sub-pathways that were strongly and consistently affected in the plasma; these included fructose, mannose, and galactose; leucine, isoleucine, and valine; diacelyglycerol; polyunsaturated fatty acid; and fatty acid monohydroxy pathways, among others [28]. Further analysis for disease and function enrichment in both the prior and current NOD datasets consistently indicated evident changes in the systemic metabolome in association with T1D onset. Notably, the analysis identified metabolic pathways with strong associations with immunological and inflammatory conditions (Figure 6).

**Figure 7:**
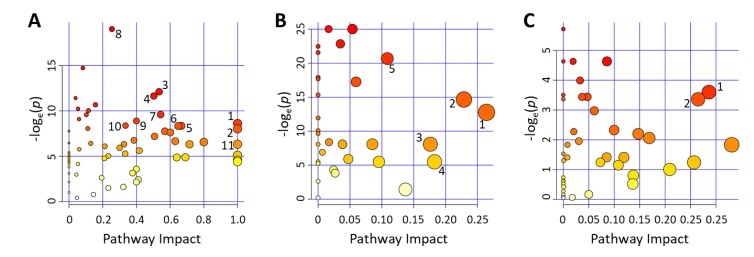
Pathway-impact analysis showing the metabolic pathways (**A**) most affected by onset of T1D in NOD mice; (**B**) associated with metabolites enriched in the aqueous humor versus plasma; and (**C**) associated with metabolites showing equal distribution in both compartments of nondiabetic mice. The shown pairwise comparisons highlight the most impacted pathways (numbered) with an Impact Factor >0.1 as identified by metabolites showing significant pathway enrichment (see Figure 4) with a corresponding *p* value < 0.05 (i.e., −log_e_(*p*) > 2.99) in quantitative enrichment tests (Global-test and Global-Ancova) performed by MetaboAnalyst. The plots in each panel are scaled independently based on the number of metabolites (see above). Color gradient corresponds to the −log_e_(*p*) value with red being the highest, and symbol size is proportional to the predicted impact factor. Numbered pathways in (**A**) correspond to 1. linoleic acid metabolism; 2. valine, leucine, and isoleucine metabolism; 3. arginine and proline metabolism; 4. glycerophospholipid metabolism; 5. beta-alanine metabolism; 6. phenylalanine metabolism; 7. sphingolipid metabolism; 8. starch and sucrose metabolism; 9. ascorbate and aldarate metabolism; 10. purine metabolism; 11. taurine and hypotaurine metabolism. Numbered pathways in (**B**) correspond to 1. taurine and hypotaurine metabolism; 2. cysteine and methionine metabolism; 3. valine, leucine, and isoleucine biosynthesis; 4. glycine, serine, and threonine metabolism; 5. phenylalanine, tyrosine, and tryptophan biosynthesis. Numbered pathways in (**C**) correspond to 1. D-Arginine and D-ornithine metabolism; 2. citrate cycle (tricarboxylic acid cycle).

Pathway-impact analysis (MetaboAnalyst) of the plasma dataset from progressors versus non-progressors (at 26 weeks of age) (Figure 7A) showed that the metabolic pathways most impacted (Impact Factor >0.1) included linoleic acid metabolism; valine, leucine, and isoleucine metabolism; and taurine and hypotaurine metabolism; whereas, those most significantly affected (−log_e_(*p*) >2.99) included starch and sucrose metabolism; arginine and proline metabolism; and glycerophospholipid metabolism (Figure 7A). Notably, there were many overlapping pathways when comparing the findings from the prior dataset with those in the present one for metabolites found to be either equally distributed or enriched in aqueous humor compared to the systemic (plasma) levels with and without diabetes. Localized metabolomics in the aqueous humor samples provided information particularly relevant to pathways involving alanine, aspartic acid, and glutamate metabolism; arginine and proline metabolism; taurine and hypotaurine metabolism; pyruvate metabolism; glycine, serine, and threonine metabolism (Figure 7B). Metabolites showing equal distribution in both compartments were mostly relevant to arginine and ornithine metabolism as well as the citrate cycle (TCA cycle) (Figure 7C). The majority of pathways identified in both datasets were affected by the onset of T1D (see Figure S5), and many of them were found to be implicated in proinflammatory conditions (Figure 6).

### 2.3. Gender-Specific Differences in the Metabolome

**Figure 8:**
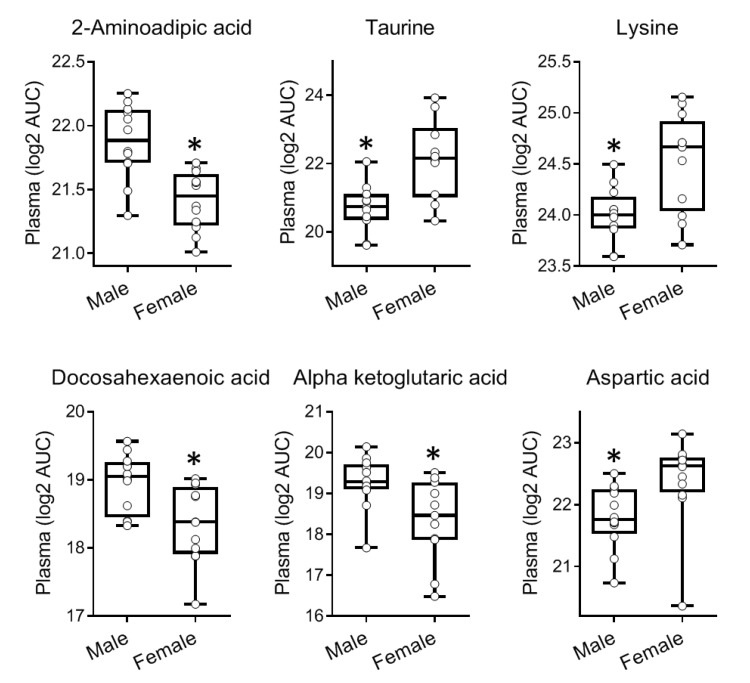
Metabolites showing significant differences in plasma levels in male versus female nondiabetic C57BL/6 mice (*n* = 12; 6M + 6F). Data are shown as Box and Whisker plots with individual datapoints corresponding to each sample shown as round symbols. Asterisk denotes *p* < 0.05 by unpaired *t*-test.

We compared in the present dataset the levels of metabolites measured in plasma samples of male and female nondiabetic C57BL/6 mice (*n* = 12 each). No such comparisons were made for NOD mice as all NOD samples were from females. Of the 122 identified metabolites, 16 showed significant differences (*p* ≤ 0.05) between males and females in plasma. The largest differences were found for 2-hydroxyvaleric acid, 2-aminoadipic acid, inosine/adenosine, myoinositol, taurine, lysine, 2-ketoleucine/ketoisoleucine, docosahexaenoic acid, O-phosphocolamine, and α-ketoglutaric acid (Figure 8). Corresponding pooled aqueous humor samples showed similar trends for several metabolites, although, no significant differences were measured likely due to the smaller number of samples (*n* = 3) (Figure S3).

Analysis of the prior dataset from the longitudinal NOD study also found many metabolites that had significantly (*p* < 0.05) different levels in males versus females (e.g., 351 out of the 676 metabolites identified in blood had significant sex-associated differences) [28]. Several of the metabolites had up to 2–3-fold differences irrespective of T1D onset (Figure S4). Examples of metabolites with the largest and most consistent differences in the blood included β-alanine, carboxyethyl-GABA, imidazole lactate, hexanoylglycine, 4-cholesten-3-one, several sphingosine derivatives, and tryptophan.

## 3. Discussion

We have previously shown that the aqueous humor provides a good representative sample of the immediate microenvironment of pancreatic islets when they are transplanted in the anterior chamber of the eye (ACE) to investigate immune responses during T1D development and islet graft rejection [23,24,35]. The current studies further demonstrate that aqueous humor samples can be collected from mice and pooled in sufficient volumes to allow localized non-targeted metabolomic studies. Although not shown here, targeted metabolomics can also be performed on aqueous humor samples from individual mice using other techniques that require nanoliter sample volumes, such as capillary zone electrophoresis and laser-induced fluorescence detection (CZE-LIFD) [36,37]. Importantly, the current studies showed that systemic metabolites relevant to pancreatic islets and diabetes were well represented in the aqueous humor; thus, providing proof-of-concept evidence for using aqueous humor samples, that may be even more enriched with islet- and immune-related metabolites after intraocular islet transplant, in targeted and non-targeted localized metabolomics approaches [23]. Although preliminary, the current non-targeted metabolomic study in nondiabetic mice notably indicated that the aqueous humor could provide enriched information on metabolic pathways relevant to inflammatory and immune diseases (Figure 7B). The results further showed that many of the pathways identified in the aqueous humor of nondiabetic mice were also significantly affected by the onset of autoimmune diabetes in the NOD mice (Figure 7). Closer analysis of the associated pathways showed that 34 out of 37 pathway-associated metabolite sets (that showed enrichment in the aqueous humor compared to plasma of the nondiabetic C57BL/6 mice) were also among the 59 pathways identified as significantly affected by T1D onset in NOD mice (Figure S5A). This is encouraging when using localized metabolomics coupled to intraocular islet transplant because longitudinally collected aqueous humor samples representative of the immediate islet microenvironment could provide direct information on the progression of auto- or allo-immune attack directed against pancreatic islets there, during progression of T1D or islet allograft rejection, respectively [23,24,38,39]. Furthermore, pathway-associated metabolite sets that were similarly represented in the aqueous humor and plasma of nondiabetic mice (without intraocular islet transplant) also showed complete overlap with those most affected by diabetes, as all 12 were among the 59 pathways affected by T1D onset in the progressor NOD mice (Figure S5B). Taken together, the above findings provided further support to the notion that aqueous humor sampling for metabolomic studies of locally enriched islet-related metabolites (with and without intraocular islet transplant) is advantageous in the identification of islet- and possibly immune-relevant metabolites. Specifically, such enriched metabolites may facilitate the discovery of reliable early biomarkers of T1D that could have been overlooked in the general circulation by prior investigations since it is not clear how well-represented the islet microenvironment is in the periphery.

As indicated in our prior longitudinal NOD study, onset of hyperglycemia profoundly altered the overall metabolic landscape (Figure 5 and Figure S2B) affecting the corresponding pathways (Figure 7), even though, hyperglycemia was partially controlled in the diabetic animals using sustained release insulin implants. For example, 3-hydroxybutyric acid showed the second largest increase (>10-fold) in blood by onset of hyperglycemia at 26 weeks of age. While fewer compounds showed relative decrease in T1D progressors, a 50-fold reduction in blood of 1,5-anhydroglucitol was measured following onset of T1D. Notably, both 3-hydroxybutyric acid and 1,5-anhydroglucitol have been used as clinical biomarkers in diabetes. 1,5-anhydroglucitol is used as a marker of glycemic control [40] and 3-hydroxybutyric acid (β-hydroxybutyric acid, BHBA; a ketone body) as an indicator of diabetic ketoacidosis due to insulin deficiency in T1D [41,42]. Notably, 1,5-anhydroglucitol had strong correlation between its plasma and aqueous humor levels (Figure 2). Furthermore, additional pathway analysis identified the pro-inflammatory cytokine TNF-α as central element in a network generated in silico (by Ingenuity Pathway Analysis software) using the prior metabolomics dataset acquired longitudinally in the T1D progressor NOD mice (Figure S6). TNF-α is widely recognized to play a key role in T1D pathogenesis and onset of hyperglycemia [43].

Monitoring changes in amino acids, including the branched-chain amino acids (isoleucine, leucine, and valine) and aromatic amino acids (tyrosine, phenylalanine, and tryptophan), and their metabolism could be of particular interest for T1D [31,44]. These specific amino acid metabolism pathways were found to have highly significant associations with future T1D incidence in a clinical study [45]. The same study suggested that fasting concentrations of these amino acids are higher in T1D progressor than in non-progressor human subjects. Consistently, our data showed branched-chain and aromatic amino acids metabolism pathways were among the most significantly impacted pathways in the NOD mice (Figure 7A). Notably, several of these amino acids including tryptophan were found to be equally distributed or enriched in the aqueous humor samples compared to plasma (Figure 1). Tryptophan metabolism is of particular interest to us as we previously identified the kynurenine and kynurenate pathways as potential biomarkers of human islet inflammation [46]. 

Other studies comparing the NOD versus NOD-E and NOD-scid mice have also found kynurenate to be significantly increased with T1D [47]. The tryptophan/kynurenine pathway is the main route of tryptophan degradation/metabolism and it generates several metabolites with various activities including on immune function [48,49,50,51]. Infections can also activate this pathway to either directly evade immune recognition/clearance or indirectly modulate immune responses [49,51,52]. Hence, our observation that tryptophan levels in the aqueous humor were well below those detected in the plasma (Figure 1B) make the aqueous humor an ideal environment to detect small changes in the concentrations of tryptophan and its metabolic derivatives after intraocular islet transplant to assess the role of tryptophan metabolism in local immune reactions within the islet microenvironment both in the context of autoimmune T1D and islet allograft rejection as well as immune tolerance [53,54,55].

Furthermore, our current and prior metabolomic studies have identified clear sex differences in the systemic metabolome both in nondiabetic C57BL/6 (Figure 8) and diabetes-prone NOD mice (Figure S4). Interestingly, the trend in sex-specific differences in C57BL/6 mice was also maintained between the plasma and aqueous humor samples for several metabolites (Figure S3), although, the number of aqueous humor samples was limited. The sex-associated differences in the metabolic profiles are of interest as there is gender bias in many autoimmune diseases with females in general being more susceptible [56,57]. While there is no significant gender bias in T1D in humans, female NOD mice exhibit higher prevalence than males (60–90% versus 20–50%, respectively) [19,22]. The specific reason for this is not clear, but it probably involves a role of sex hormones [56]. Moreover, contrary to the expectation that non-progressor female NOD mice would have metabolic profiles similar to those of the less diabetes-prone male counterparts, we could not establish a relationship between hyperglycemia onset (or lack thereof) and the observed metabolic differences between male and female NOD mice. Instead, sex-dependent differences in the NOD metabolome were consistent irrespective of T1D onset (Figure S4) [28].

In summary, the current studies demonstrated the feasibility of localized metabolomics in aqueous humor samples where islet-/diabetes- and sex-associated metabolic differences were representative of those in the general circulation. Importantly, longitudinal access to the aqueous humor, where islet-related metabolites are equally distributed or enriched (with or without intraocular islet transplant), enables localized metabolomics for the identification of metabolites and associated pathways that could be affected during progression of autoimmune T1D or rejection of islet allografts. Thus, localized metabolomics may facilitate the discovery of reliable early metabolic biomarkers or biomarker-signatures that could otherwise be overlooked in the general circulation.

## 4. Materials and Methods

### 4.1. Animal Care and Treatment

All animal studies were approved by the University of Miami Institutional Animal Care and Use Committee (IACUC). Animal procedures were conducted according to the guidelines of the Committee on Care and Use of Laboratory Animals, Institute of Laboratory Animal Resources (National Research Council, Washington DC). Animals were housed in micro-isolated cages in Virus Antibody Free (VAF) rooms with free access to autoclaved food and water at the Department of Veterinary Resources of the University of Miami. Mice were obtained from the Jackson Laboratory (Bar Harbor, ME, USA). In the present study, we performed a GC-MS-based metabolomics study using blood (plasma) samples collected from 24 C57BL/6 mice (12M + 12F) and 21 NOD female mice (of which 3 were diabetic/progressors). Corresponding aqueous humor samples were also analyzed in parallel. The aqueous humor samples were pooled from groups of 4 animals to have sufficient volume (~50 μL) for the non-targeted GC-MS metabolomics. Prior studies in blood/plasma samples from progressor and non-progressor NOD mice were conducted as previously described in detail [28].

### 4.2. Sample Collection

Aqueous humor samples were collected from the anterior chamber of both eyes from each mouse by direct aspiration from the ACE using glass micropipettes with 40–60 μm tip diameter under general anesthesia. Aqueous humor from 4 mice was pooled to yield the ~50 μL samples. Parallel blood samples were obtained from the same mice immediately after aqueous humor collection through cardiac puncture followed by euthanasia. Blood was collected into purple-top EDTA Vacutainers (BD Biosciences; San Jose, CA, USA) and centrifuged immediately to separate the plasma. Plasma was transferred into acid-cleaned small 1.5 mL non-stick surface microcentrifuge tubes (VWR; Radnor, PA, USA) and frozen at −80 °C for further analysis.

### 4.3. GC-MS-Based Metabolomics Analysis

Non-targeted metabolomics of parallel plasma and aqueous humor samples were conducted in the Metabolomics Laboratory of the Duke Molecular Physiology Institute (DMPI) via gas chromatography/electron-ionization mass spectrometry (GC/ei-MS) using the method from [58] but with some differences, as described below. Both sample types were extracted by the addition of methanol spiked with perdeuterated myristic acid standard (used for adjusting GC column pressure for consistent retention time), dried by SpeedVac, and derivatized by methoximation and trimethylsilylation. A process blank consisting of no sample, but processed similarly, was additionally included in the batch run for tracking impurities. All samples were run on a 7890B GC-5977B ei-MS (Agilent Corporation; Santa Clara, CA, USA) with MS scans set broadly from *m/z* 50 to 600 during a GC heat ramp spanning 60 to 325 °C. Deconvoluted spectra were annotated as metabolites using an orthogonal approach incorporating both the GC retention time (RT) and the MS fragmentation pattern. Peak annotation was based primarily on an RT-locked spectral library of metabolites, built upon the Fiehn GC/MS Metabolomics RTL Library [59]. In this discovery protocol, chromatographic features were evaluated as log-2 transformed areas under the curve (log_2_ AUC) to represent feature abundance. Additional metabolite features that could not be annotated from the library were found by SpectConnect [60] matching spectral similarities between samples.

Prior metabolomics analysis in progressor versus non-progressor NOD mice had been conducted by LC-MS methods, as previously described in detail [28].

### 4.4. Statistical Analysis

Standard statistical analyses including *t*-tests and correlations were performed in GraphPad Prism v8.2 (GraphPad; La Jolla, CA, USA, RRID:SCR_002798). Data were curated for metabolites identified in a minimum of 6 samples and then used for the visualization of associated interaction networks and biological pathways using MetaboAnalyst (https://www.metaboanalyst.ca; RRID:SCR_015539) applying the default settings for data processing and median-normalization [32] as well as Ingenuity Pathway Analysis software (Qiagen Bioinformatics; Redwood City, CA, USA; https://www.qiagenbioinformatics.com/products/ingenuity-pathway-analysis; RRID:SCR_008653) [61]. The input for these analyses consisted of matrices of log_2_ AUC values of metabolites expressed as Human Metabolome Database (HMDB) identifiers. Approaches used in MetaboAnalyst involved the Enrichment Analysis and Pathway Analysis modules. The option chosen for the first module (Enrichment Analysis) was the concentration table known as quantitative enrichment analysis (QEA). Data was normalized by median and auto scaled (mean-centered and divided by the standard deviation of each metabolite). The metabolite set library selected was pathway-associated metabolite sets (SMPDB) using all the compounds of the selected metabolite set library as reference metabolome. In Ingenuity Pathway Analysis, we used the concentration table modality applying the same type of data normalization as described before. The component selected for pathway enrichment analysis was the global test, whereas for pathway topology analysis, it was the relative betweenness centrality. The pathway library of choice was the *Mus musculus* (SMPDB), where all the compounds served as reference metabolome.

## Data Availability

The datasets generated and/or analyzed during the current study are available from the corresponding author upon reasonable requests.

## Supplementary Materials

The following supplementary materials are available online at https://www.mdpi.com/2218-1989/9/10/207/s1, Table S1: Complete list of identified metabolites (listed alphabetically) quantified in the present GC-MS-based study of plasma and aqueous humor levels with their CAS, HMDB, and KEGG identifiers. Figure S1. Heatmap analysis of metabolite levels identified in parallel aqueous humor and corresponding plasma samples as in Figure 1 but shown separately for pooled samples obtained from normoglycemic C57BL/6 and NOD mice *(n* = 6 and 4, respectively). Figure S2: Time profile of T1D onset and corresponding changes in the blood level of the most significantly altered metabolites identified in a longitudinal NOD study. Figure S3: Metabolites that showed the same trend in their sex-associated differences in the aqueous humor as in their plasma levels, where they had significant differences between the male and female nondiabetic C57BL/6 mice. Figure S4: Selected metabolites among those consistently showing the largest significant differences between male and female NOD mice. Figure S5. Venn diagrams showing the overlap between metabolic pathways. Figure S6. TNF-α pathway networks shown as a representative of the networks identified by autoimmune and inflammatory disease analysis (Ingenuity Pathway Analysis).
