# Supplementary material for: Feasibility of Localized Metabolomics in the Study of Pancreatic Islets and Diabetes
Source: Metabolites. 2019 Sep 29;9(10):207. doi: 10.3390/metabo9100207 (PMC6835460; doi:10.3390/metabo9100207)
Supplement: Supplementary file 1 [file metabolites-09-00207-s001.pdf]

Article

Feasibility of Localized Metabolomics in the Study of Pancreatic Islets and Diabetes

Oscar Alcazar[1], Luis F. Hernandez[1], Ashley Tschiggfrie[1], Michael J. Muehlbauer[2], James R. Bain[2], Peter Buchwald[1,3]* and Midhat H. Abdulreda[1,4,5,6]*

Supplementary Materials

Table S1: Complete list of identified metabolites (listed alphabetically) quantified in the present GC-MS-based study of plasma and aqueous humor levels with their CAS, HMDB, and KEGG identifiers.

Figure S1. Heatmap analysis of metabolite levels identified in parallel aqueous humor and corresponding plasma samples as in Figure 1 but shown separately for pooled samples obtained from normoglycemic C57BL/6 and NOD mice (*n*=6 and 4, respectively).

Figure S2: Time profile of T1D onset and corresponding changes in the blood level of the most significantly altered metabolites identified in a longitudinal NOD study.

Figure S3: Metabolites that showed the same trend in their sex-associated differences in the aqueous humor as in their plasma levels, where they had significant differences between the male and female nondiabetic C57BL/6 mice.

Figure S4: Selected metabolites among those consistently showing the largest significant differences between male and female NOD mice.

Figure S5. Venn diagrams showing the overlap between metabolic pathways.

Figure S6. TNF-α pathway networks shown as a representative of the networks identified by autoimmune and inflammatory disease analysis (Ingenuity Pathway Analysis).

Supplementary Tables

Table S1: Complete list of identified metabolites (listed alphabetically) quantified in the present GC-MS-based study of plasma and aqueous humor levels with their CAS, HMDB, and KEGG identifiers.

Metabolite	CAS	HMDB	KEGG
1,2-Propanediol	57-55-6	HMDB01881	C00583
1,5-Anhydroglucitol	154-58-5	HMDB02712	C07326
1-Monoolein	111-03-5		
2-Aminoadipic acid	542-32-5	HMDB00510	C00956
2-Hydroxybutyric acid	565-70-8	HMDB00019	C00141
2-Hydroxyglutaric acid	13095-48-2	HMDB00694	C02630
2-Hydroxyisobutyric acid	594-61-6	HMDB00190	C01432
2-Hydroxypyridine	142-08-5	HMDB11749	C02502
2-Hydroxyvaleric acid	617-31-2	HMDB01863	
2-Ketoleucine/ketoisoleucine	816-66-0	HMDB00695	C00233
2-Ketovaline	759-05-7	HMDB00161	C00041
3-(3-Hydroxyphenyl)propionic acid	621-54-5	HMDB00375	C11457
3-Hydroxyindole	480-93-3	HMDB04094	
3-Indolelactic acid/tryptophan	1821-52-9	HMDB00671	C02043
5-Hydroxylysine/dopamine	1190-94-9	HMDB00450	C16741
6-Deoxyhexose	3615-41-6	HMDB00849	C00507
Acetylsalicylic acid/Salicylic acid	50-78-2	HMDB01879	C01405
Adenosine/Inosine	58-61-7	HMDB00050	C00212
Alanine	56-41-7	HMDB00115	C00160
Aldopentoses	147-81-9	HMDB00646	C00259
Allantoin	97-59-6	HMDB00462	C01551
alpha Ketoglutaric acid	328-50-7	HMDB00208	C00026
alpha-Monopalmitin	542-44-9	HMDB05356	
alpha-Monostearin	123-94-4	HMDB31075	D01947
alpha-Tocopherol	10191-41-0		
Aminomalonic acid	1068-84-4	HMDB01147	C00872
Arachidic acid/ 1-Heneicosanol	506-30-9	HMDB02212	C06425
Arginine	74-79-3	HMDB00517	C00062
Asparagine	70-47-3	HMDB00168	C00152
Aspartic acid	56-84-8	HMDB00191	C00049
Benzoic acid	65-85-0	HMDB01870	C00180
beta-Alanine	107-95-9	HMDB00056	C00099
beta-Hydroxybutyric acid	300-85-6	HMDB00357	
beta-Monopalmitin	23470-00-0	HMDB11533	
beta-Monostearin	31566-31-1	HMDB11535	D01947
Campesterol	474-62-4	HMDB02869	C01789
Cholesterol	57-88-5	HMDB00067	C00187
Citramalic acid	597-44-4	HMDB00426	
Citric acid/isocitric acid	5949-29-1		C12649
Cystine	56-89-3	HMDB00192	C00491
Docosahexaenoic acid	6217-54-5	HMDB02183	
Eicosapentaenoic acid	10417-94-4	HMDB01999	C06428
Erythronic acid	13752-84-6	HMDB00613	
Erythrose/Threose	533-49-3		
Ethanolamine	141-43-5	HMDB00149	C00189
Fructose or similar ketohexose	57-48-7	HMDB00660	C10906
Fumaric acid	110-17-8	HMDB00134	C00122

Metabolite	CAS	HMDB	KEGG
Gluconic acid or similar sugar acid	526-95-4	HMDB00625	C00257
Glucose and other aldohexoses	50-99-7	HMDB00516	C00221
Glutamic acid	56-86-0	HMDB00148	C00025
Glutamine	56-85-9	HMDB00641	C00064
Glyceric acid	473-81-4	HMDB00139	C00258
Glycerol	56-81-5	HMDB00131	C00116
Glycerol 1-phosphate	34363-28-5		
Glycine	56-40-6	HMDB00123	C00037
Glycolic acid	79-14-1	HMDB00729	
Heptadecanoic acid/Octadecanol	506-12-7	HMDB02259	
Hippuric acid	495-69-2	HMDB00714	C01586
Hydrocinnamic acid	501-52-0	HMDB00764	C05629
Hydroxyprolines	51-35-4	HMDB00725	C01157
Hypotaurine	300-84-5	HMDB00965	C00519
Hypoxanthine	68-94-0	HMDB00157	C00262
Inosine/Adenosine	58-63-9	HMDB00195	C00294
Isoleucine	443-79-8	HMDB00172	C00407
Lactic acid	79-33-4	HMDB00243	C00022
Lactose or similar disaccharide	63-42-3	HMDB00186	C00243
Leucine	61-90-5	HMDB00687	C00123
Linoleic acid	60-33-3	HMDB00673	C01595
Lysine	56-87-1	HMDB00182	C00047
Malic acid	617-48-1	HMDB00744	C00711
Methionine	63-68-3	HMDB00696	C01733
Methionine sulfoxide	3226-65-1	HMDB02005	C02989
Methyl linoleate	112-63-0	HMDB34381	
Methyl palmitate	112-39-0		C16995
Methyl stearate	112-61-8	HMDB34154	
Methylglutamic acid	6753-62-4		
Myoinositol	87-89-8	HMDB00211	C00137
Myristic acid or Pentadecanol	629-76-5	HMDB13299	
N-Acetylaspartic acid	997-55-7	HMDB00812	
N-Methylalanine/2-Aminobutanoic acid/N-Ethylglycine	100-61-8		C02299
Nonanoic acid	112-05-0	HMDB00847	C01601
Oleic acid	112-80-1	HMDB00207	C00712
O-Methylphosphate	812-00-0		
O-Phosphocolamine	1071-23-4	HMDB00224	C00346
Ornithine	70-26-8	HMDB00214	C00077
Palmitic acid	57-10-3	HMDB00220	C00249
Palmitoleic acid	373-49-9	HMDB03229	C08362
Pantothenic acid	137-08-6		D01082
Pentitols	488-82-4	HMDB00568	C01904
Pentonic acids	4172-43-4		
Phenylalanine	63-91-2	HMDB00159	C02057
Phosphoric acid	7664-38-2	HMDB02142	C00009
p-Hydroxyphenyllactic acid	6482-98-0	HMDB00755	C03672
Pinitol or similar methoxyinositol	484-68-4		
Pipecolic acid	535-75-1	HMDB00070	
Proline	147-85-3	HMDB00162	C00148
Pseudouridine	1445-07-4	HMDB00767	C02067

Metabolite	CAS	HMDB	KEGG
Putrescine	110-60-1	HMDB01414	C00134
Pyruvic acid	127-17-3	HMDB11749	C02502
Sarcosine	107-97-1	HMDB00008	
Serine	56-45-1	HMDB00187	C00716
Serotonin	50-67-9	HMDB00259	C00780
Spermidine	124-20-9	HMDB01257	C00315
Stearamide	124-26-5	HMDB34146	C13846
Stearic acid	57-11-4	HMDB00827	C01530
Succinic acid	110-15-6	HMDB00254	C00042
Sucrose and similar disaccharides	57-50-1	HMDB00258	C00089
Taurine	107-35-7	HMDB00251	C00245
Threitol/Erythritol	6968-16-7		
Threonic acid	3909-12-4	HMDB00943	C01620
Threonine	72-19-5	HMDB00167	C00188
Tiglic acid	80-59-1	HMDB01470	C08279
Tryptamine/Norepinephrine	61-54-1	HMDB00303	C00398
Tryptophan	73-22-3	HMDB00929	C00806
Tyrosine	60-18-4	HMDB00158	C00082
Uracil	66-22-8	HMDB00300	C00106
Urea	57-13-6	HMDB00294	C00086
Uric acid	69-93-2	HMDB00289	C00366
Uridine	58-96-8	HMDB00296	C00299
Valine	72-18-4	HMDB00883	C00183
Xanthine	69-89-6	HMDB00292	C00385

Supplementary Figures

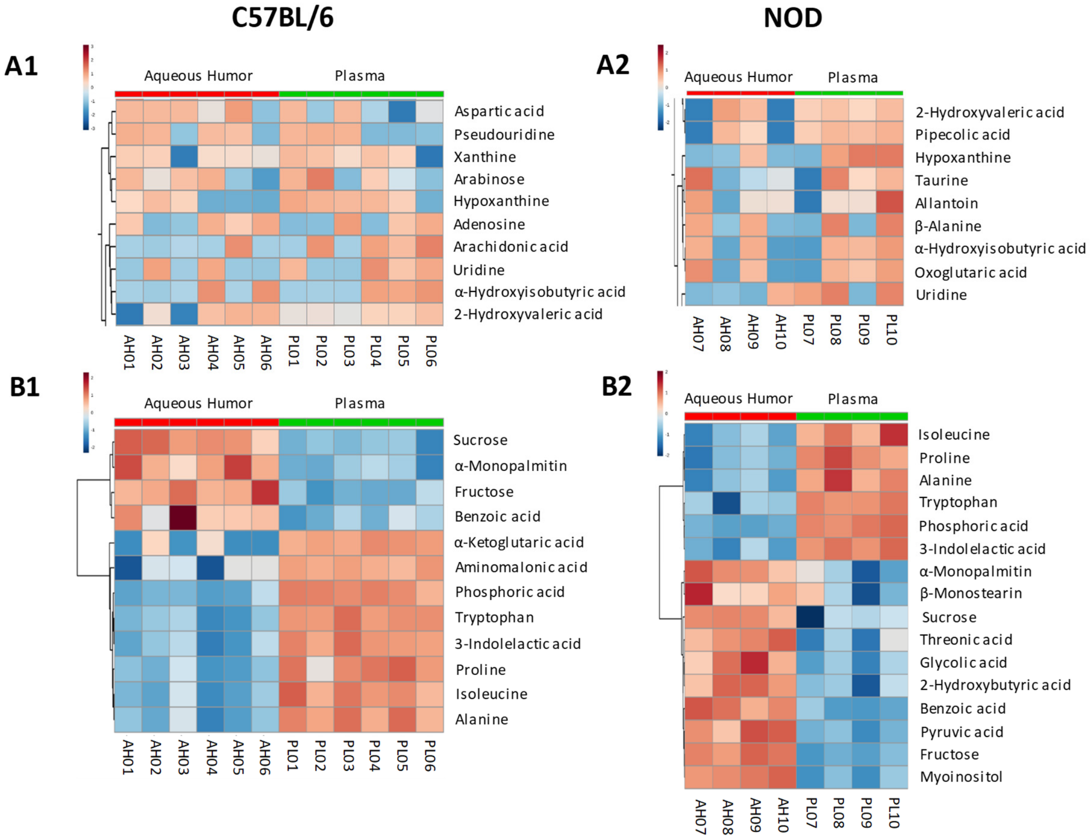

Figure S1. Heatmap analysis of metabolite levels identified in parallel aqueous humor and corresponding plasma samples as in Figure 1 but shown separately for pooled samples obtained from normoglycemic C57BL/6 and NOD mice (*n*=6 and 4, respectively). Data shown are for metabolites that are most equally (**A1**, **A2**) and (**B1**, **B2**) differently distributed between the aqueous humor and plasma.

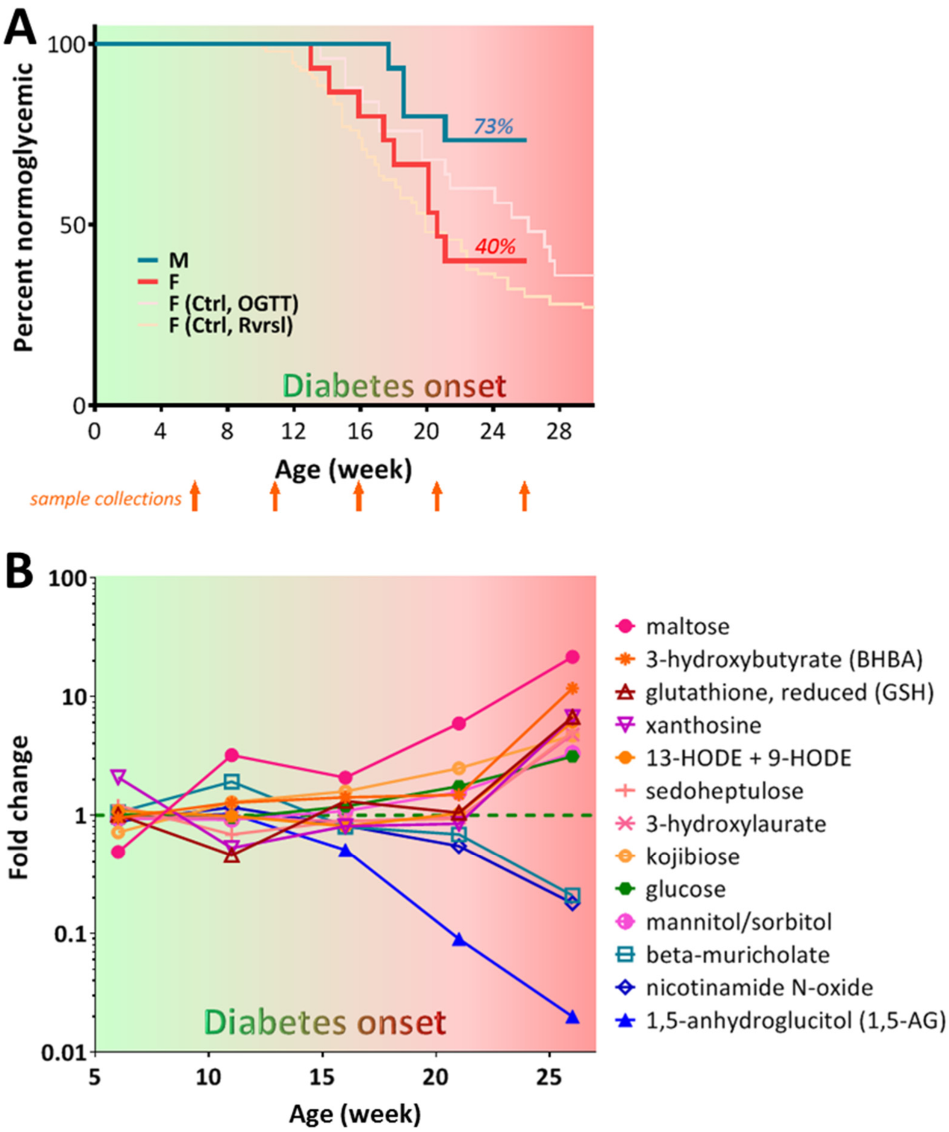

Figure S2. Time profile of T1D onset and corresponding changes in the blood level of the most significantly altered metabolites identified in the longitudinal NOD study [28]. (**A**) Kaplan-Meier survival curves showing the normoglycemia rate in these NOD mice. Data for male (M; *n*=15) and female (F; *n*=15) mice are shown separately in blue and red, respectively. Sample collections times are denoted with orange arrows. Lighter lines indicate onset rates obtained in two other studies by us with female NODs for comparison [33,34]. (**B**) Longitudinal time-profile of the metabolites showing the largest fold-change in diabetic *versus* control mice in blood samples at week 26 (representative metabolites with >4-fold change and $p<0.05$). Data shown on log scale to incorporate compounds that show increase as well as decrease *versus* nondiabetic control. Background color indicates diabetes onset as green fading to red; all progressors were confirmed diabetic by 26 weeks of age.

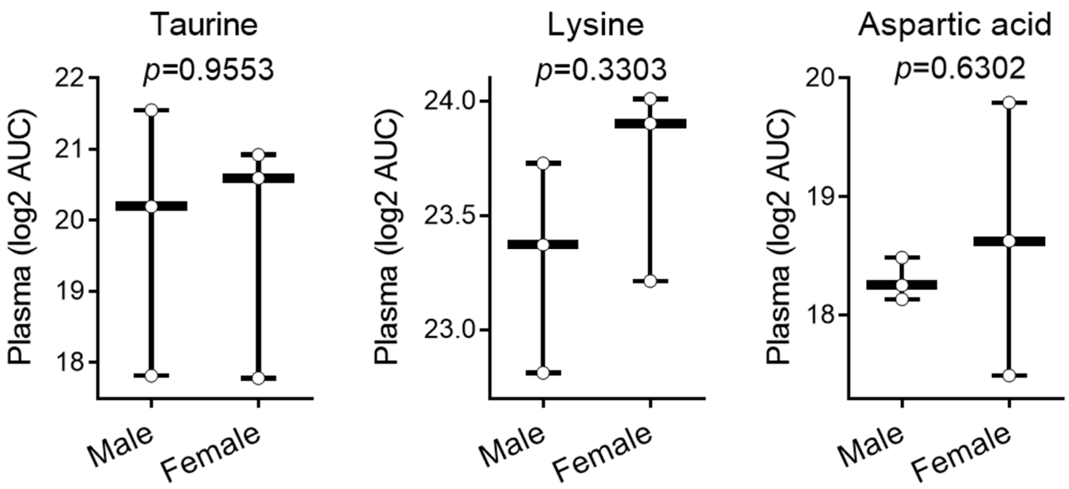

Figure S3. Metabolites that showed the same trend in their sex-associated differences in the aqueous humor as in their plasma levels, where they had significant differences between the male and female nondiabetic C57BL/6 mice (see **Figure 8**). Data shown as Box and Whiskers plots (n=3 pooled from 12 mice; 4 mice each).

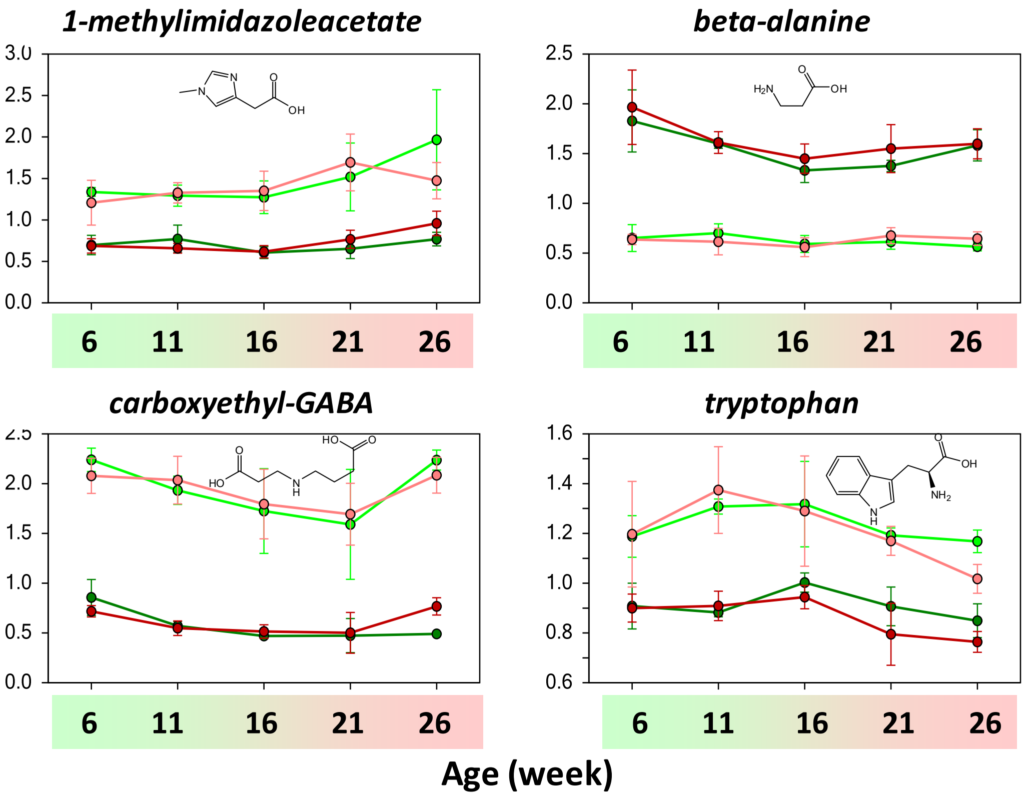

Figure S4. Selected metabolites among those consistently showing the largest significant differences between male and female NOD mice [28]. Line profiles showing change in time are color coded for T1D progressors (red) *versus* non-progressors (green) with males in darker and females in lighter hues.

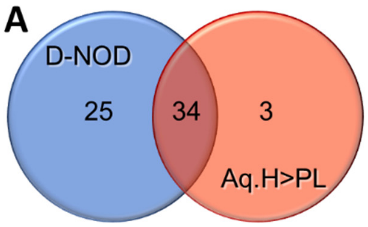

Figure S5. Venn diagrams showing the overlap between metabolic pathways corresponding to metabolite sets that were significantly affected by T1D onset in NOD mice (D-NOD) and pathways corresponding to metabolite sets that were found (**A**) to be significantly enriched or (**B**) equally distributed when comparing parallel aqueous humor (Aq.H) and plasma (PL) samples from the same nondiabetic mice.

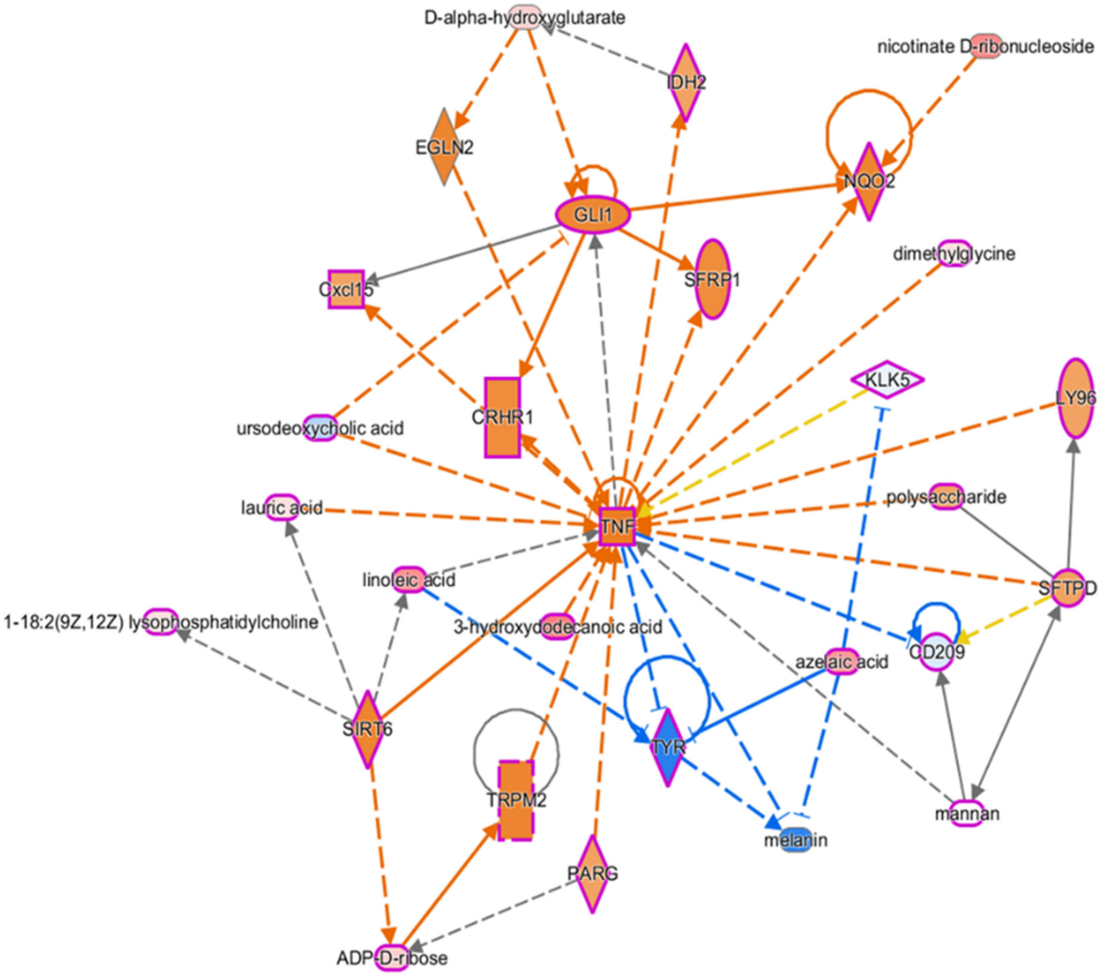

Figure S6. An illustrative pathways network identified by autoimmune and inflammatory disease analysis (Ingenuity Pathway Analysis software) as being one of the most affected pathways by T1D onset in NOD mice. This pathway has tumor necrosis factor-alpha (TNF-α) as its central node. TNF-α is well-known to play important roles in T1D pathogenesis leading to onset of hyperglycemia [43]. Network elements are represented by various shape symbols and colors. Marker colors: orange denotes predicted activation and blue predicted inhibition. Connecting lines: orange denotes activation, blue inhibition, yellow findings that are inconsistent with the state of the downstream molecule, and gray means not predicted. Marker shapes: horizontal-oval denotes transcription regulator, vertical-oval - transmembrane receptor, diamond - enzyme, square - cytokine, vertical-rectangle - G-protein coupled receptor, broken-lined vertical-rectangle - ion channel, and horizontal-diamond - peptidase. Abbreviations: CD209 – dendritic cell-specific ICAM-3-grabbing non-integrin 1, CRHR1 – corticotropin releasing hormone receptor 1, Cxcl15 – chemokine (C-X-C motif) ligand 15, EGLN2 – Egl-9 family hypoxia inducible factor 2, GLI1 – GLI family zinc finger 1, IDH2 – isocitrate dehydrogenase 2 ($NADP^+$) mitochondrial, KLK5 – kallikrein related peptidase 5, LY96 – lymphocyte antigen 96, NQO2 – NAD(P)H quinone dehydrogenase 2, PARG – poly(ADP-ribose) glycohydrolase, SFRP1 – secreted Frizzled related protein 1, SFTPD – surfactant protein D, SIRT6 – sirtuin 6, TNF – tumor necrosis factor, TRPM2 – transient receptor potential cation channel subfamily M member 2, TYR – tyrosinase.
